# ‘I am treated well if I adhere to my HIV medication’: putting patient–provider interactions in context through insights from qualitative research in five sub-Saharan African countries

**DOI:** 10.1136/sextrans-2016-052973

**Published:** 2017-07-23

**Authors:** Ken Ondenge, Jenny Renju, Oliver Bonnington, Mosa Moshabela, Joyce Wamoyi, Constance Nyamukapa, Janet Seeley, Alison Wringe, Morten Skovdal

**Affiliations:** 1 Kenya Medical Research Institute (KEMRI), Centre for Global Health Research, Kisumu, Kenya; 2 Department of Population Health, London School of Hygiene & Tropical Medicine, London, UK; 3 Malawi Epidemiology and Intervention Research Unit, Karonga, Malawi; 4 Africa Health Research Institute, KwaZulu-Natal, South Africa; 5 University of KwaZulu-Natal, Durban, South Africa; 6 National Institute of Medical Research, Mwanza, United Republic of Tanzania; 7 Manicaland Centre for Public Health Research, Biomedical Research and Training Institute, Harare, Zimbabwe; 8 Department of Public Health, University of Copenhagen, Copenhagen, Denmark

**Keywords:** AFRICA, ANTERETROVIRAL THERAPY, HIV CLINICAL CARE, QUALITATIVE RESEARCH

## Abstract

**Objectives:**

The nature of patient–provider interactions and communication is widely documented to significantly impact on patient experiences, treatment adherence and health outcomes. Yet little is known about the broader contextual factors and dynamics that shape patient–provider interactions in high HIV prevalence and limited-resource settings. Drawing on qualitative research from five sub-Saharan African countries, we seek to unpack local dynamics that serve to hinder or facilitate productive patient–provider interactions.

**Methods:**

This qualitative study, conducted in Kisumu (Kenya), Kisesa (Tanzania), Manicaland (Zimbabwe), Karonga (Malawi) and uMkhanyakude (South Africa), draws upon 278 in-depth interviews with purposively sampled people living with HIV with different diagnosis and treatment histories, 29 family members of people who died due to HIV and 38 HIV healthcare workers. Data were collected using topic guides that explored patient testing and antiretroviral therapy treatment journeys. Thematic analysis was conducted, aided by NVivo V.8.0 software.

**Results:**

Our analysis revealed an array of inter-related contextual factors and power dynamics shaping patient–provider interactions. These included (1) participants’ perceptions of roles and identities of ‘self’ and ‘other’; (2) conformity or resistance to the ‘rules of HIV service engagement’ and a ‘patient-persona’; (3) the influence of significant others’ views on service provision; and (4) resources in health services. We observed that these four factors/dynamics were located in the wider context of conceptualisations of power, autonomy and structure.

**Conclusion:**

Patient–provider interaction is complex, multidimensional and deeply embedded in wider social dynamics. Multiple contextual domains shape patient–provider interactions in the context of HIV in sub-Saharan Africa. Interventions to improve patient experiences and treatment adherence through enhanced interactions need to go beyond the existing focus on patient–provider communication strategies.

## Introduction

Over the past decade, unprecedented efforts have been made to bring antiretroviral therapy (ART) to almost 14 million people in sub-Saharan Africa, despite the substantial challenges posed by fragile health systems.^1^ The increase in ART coverage in the region can be partly attributed to the adoption of policies to expand testing opportunities, decentralisation of HIV services, and promotion of task shifting and simplification of care and treatment protocols.^2^ The culmination of such policies has led to more counsellors, nurses and lay health workers who are involved in diagnosing and managing HIV infection.^3^

Much has been written about patient–provider interactions,^4 5^ often guided by the sociological and psychological dynamics that arise from representations of good and bad, easy and difficult, and desirable and undesirable patients.^6^ Such research is often from the perspectives of healthcare providers, who reflect on what kind of patients are most likely to benefit from a therapeutic relationship with healthcare providers. Needless to say, such studies say more about the biases,^7 8^ communication styles^9^ and day-to-day frustrations^10^ of healthcare providers than patient behaviours per se. There have been calls for greater involvement of patients’ perspectives on patient–provider interactions.^4^

A growing number of qualitative studies have emerged to detail the nature of patient–provider interactions in the context of HIV, and how these can either promote or hinder engagement with HIV services.^11–13^ In Tanzania, for example, Gourlay and colleagues^12^ found (dis)trust, level of communication and (dis)respect influenced interactions and engagement with prevention of mother-to-child transmission services. In Zimbabwe, Campbell and colleagues^13^ found social representations of ‘good’ and ‘bad’ patient persona shaped interactions between patients and providers, with those who conformed to the norms of a ‘good patient persona’ experiencing productive and health-enabling patient–nurse relationships, and those who did not being likely to experience a breakdown in their relationship. A growing number of studies also demonstrate the importance of patient experiences on HIV treatment adherence and health outcomes.^14–18^

Much of the existing research focuses on communication and interactions during consultations, and explores how these factors influence patient–provider relationships, either with beneficial or detrimental effects on their engagement in care and treatment services. To date, there is a paucity of qualitative research exploring the array of contextual factors and dynamics that shape patient–provider interactions. This paper uses data from a multicountry qualitative study to explore the contextual factors and dynamics that shape patient–provider interactions, and in turn influence patient engagement in HIV programmes.

### Theoretical perspectives

The emergence of chronic conditions such as HIV has necessitated a shift in clinical medicine, provoking new questions and offering new challenges to patient–provider relations. Specifically, the increasing requirements for patients’ long-term/lifelong adherence to medication for chronic conditions have tilted the trend from a provider-centred to a patient-focused approach.^19^ With this shift in mind, we draw on phenomenological approaches to explore the complex yet critical relationships between people living with HIV (PLHIVs) and providers that underpin patients’ engagement with HIV care and treatment in five sub-Saharan African settings.

Phenomenological approaches are based on a paradigm of personal knowledge and subjectivity, and emphasise the importance of personal perspective and interpretation.^20^ As such they are powerful for understanding subjective experience, gaining insights into people’s motivations and actions, and challenging underlying assumptions and conventional wisdom. An interpretive dimension to the phenomenological approach enables it to be used to inform, support or challenge policy and practice.^21 22^

## Methods

The data for this analysis are drawn from a multisite qualitative study, ‘the Bottlenecks study’, which aimed to explore how contextual, social and health systems factors influence the engagement of PLHIVs with HIV care and treatment in seven health and demographic surveillance sites in sub-Saharan Africa. Each setting was rural in nature and experiencing a generalised HIV epidemic. Health services including those for HIV were generally government-run and delivered through small health facilities. The methods for the overall study and study settings are described in detail in the methods supplement  found in the editorial at http://dx.doi.org/10.1136/sextrans-2017-053172 23.

Ethical approval for the study was granted by the London School of Hygiene & Tropical Medicine and the relevant ethics boards at each of the study settings. Written and informed consent for participation in the study was granted from all participants on the condition of anonymity.

### Study location and participants

A total of 278 individual in-depth interviews were conducted between December 2015 and February 2016 among PLHIVs of both sexes and across a range of ages. PLHIVs were sampled with different care and treatment histories, relatives of persons deceased from HIV and healthcare providers in each study site (table 1).

**Table 1:** Participants’ distribution

Country	HDSS	HCW	Persons living with HIV			Family member of the deceased	Country total
			Never ART	On ART	LTFU		
Kenya	Kisumu	8	10	13	8	11	50
Tanzania	Kisesa	7	13	14	4	6	44
Malawi	Karonga	5	9	27	4	6	51
Zimbabwe	Manicaland	4	16	35	8	6	69
South Africa	uMkhanyakude	19	16	17	6	6	64
Total		43	64	106	30	35	278

ART, antiretroviral therapy; HCW, healthcare worker; HDSS, health and demographic surveillance sites; LTFU, lost to follow-up from an HIV clinic for >90 days.

PLHIVs were sampled from health and demographic surveillance databases or from HIV clinic records. Family members of PLHIVs who had recently died were identified using verbal autopsy data sets, ensuring that only family members who knew the status of the deceased were sampled. Healthcare providers were purposively sampled to include participants with a variety of roles in HIV care, and were invited to participate by the study coordinators.

### Data collection and analysis

Semistructured interview guides containing open-ended questions on experiences of the provision or receipt of HIV services were translated into local languages. Same-sex interviews were administered in private by trained, local fieldworkers who were not previously known to the participants. The interviews were conducted either in health facilities or in participants’ homes. Interviews were audio-recorded, transcribed verbatim and non-English transcripts were translated using a meaning-based approach. Interviewers kept summary notes based on non-structured observations during their fieldwork and held daily debriefing sessions with the local study coordinators to review their field experiences and emerging ideas from the case narratives. Broad coding was applied by the team leader in each country using a framework approach.^24^ A second level of inductive analysis was conducted by the lead author on all nodes pertaining to patient–provider interactions as well as the overall interview summaries.

The thematic data analysis followed phenomenological procedures described by Hycner^22^ and was completed using NVivo V.8.0.

## Results

**Figure 1:**
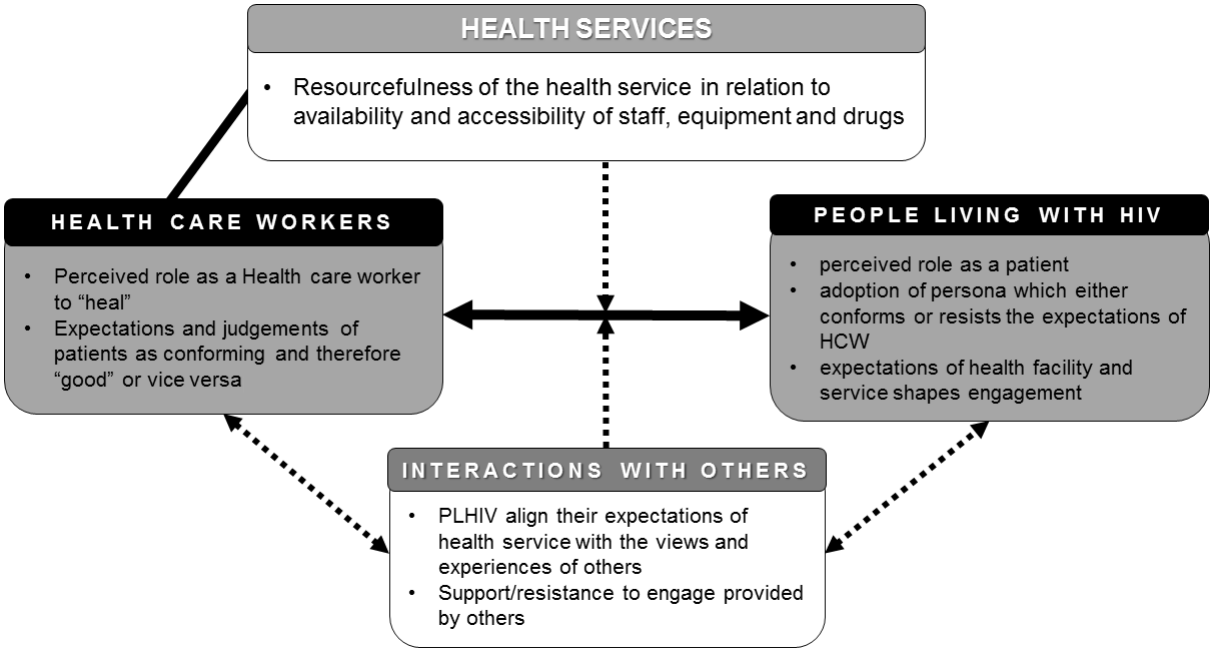
Schematic representation of the contextual factors and power dynamics shaping patient–provider interactions in the context of HIV care and treatment in sub-Saharan Africa. HCW, healthcare worker; PLHIV, people living with HIV.

Our analysis revealed an ecology of factors and dynamics shaping patient–provider interactions. These foundational factors include (1) perceived roles and identities; (2) conformity or resistance to the ‘patient-persona’; (3) how significant others talk about healthcare providers; and (4) the resources in health services (see figure 1).

### Perceived roles and identities

Power asymmetries between healthcare providers and their patients were reflected in how both parties viewed themselves and each other. Such asymmetries appeared to establish order and directed codes of conduct in patient–provider interactions. Some PLHIVs experienced forms of abuse of power by providers, without recourse to remedial action, rendering them helpless and powerless. Some also reported resigning their fate to the providers given their “unflattering chronic health state, suggesting that it was only the doctors who could help them” and that “there was nothing they [PLHIV] could do to change whatever the Lord had given them [HIV].” Some PLHIVs recounted experiences of long waiting times in which they described themselves as being at the mercy of the providers.

*Of course there is no [fair] treatment…they would just be chatting while people continue gathering. You would just wait…what can you do given that you’ve come to collect the medicines? You just have to sit down and wait Yeah, there would be a lot of people and eventually you get tired. (PLHIV, on ART, East Africa)*

In describing their role in HIV care and treatment, many health workers perceived themselves as ‘healers’ and providers of help with the ability and mandate to treat opportunistic infections, provide drugs, manage any adverse reactions and address adherence challenges:

*I make people who are being looked upon as…a foregone case come back to life and move on with their daily activities…you find that they recover and they go back to their jobs and they carry on with their lives despite being HIV-positive. (Healthcare worker (HCW), East Africa)*

A reflection of this power hierarchy was also seen among the views of PLHIVs. In many cases, PLHIVs concurred with the providers, which further exacerbated the effects of patient–provider power differences:

*The hospital personnel are the ones who handle the situation of every patient and know the type of medicine to give depending on that disease. Here in the village we don’t know anything about how this medicine can make us to get healed from this disease. (Family member of a PLHIV who had died from HIV, Southern Africa)*

Such instantiations of ‘self’ and ‘other’ provide context for how patients and health workers position themselves for health-enabling patient–provider interactions, or, as we will discuss next, how patients ought to position themselves in relation to providers to optimise the health-enabling potential of their interactions.

### Conformity or resistance to the ‘patient-persona’

PLHIVs had mixed experiences with the health services. While some reported meeting ‘supportive’ and ‘understanding’ providers willing to share and listen to them, others reported being ‘harassed’ by providers into initiating care without being ‘listened to’ and given time to absorb and accept their condition. They reported being rebuked by providers, ‘talked to carelessly’ and in some cases being ‘sent away’ from the facility, often culminating in a decision to disengage with facility care.

*I was very free with the nurse that I had to begin with, but she was transferred after three months. The next one was harassing people; she did not know how to talk to the clients and that made many people leave. (PLHIV, lost to follow-up, East Africa)*

A patient in southern Africa illustrated the conditional nature of the relationship:

*I am treated well if I adhere to my HIV medication. They would encourage me. There is no single day that they harassed me. (PLHIV, on ART, Southern Africa)*

Similarly, many providers reported that they appreciated patients who were proactive, did not question ‘the rules given to them’, ‘kept their clinic appointment dates’, ‘came to the clinic on time’ and ‘took their medication’. The providers perceived such patients as ‘good patients’ who they appreciated as worthy of their help, and gave them privileges.

*We tell them, don’t miss your clinic…if you abide by the rules which have been set for you, you will lead a comfortable life…One who listens to the doctor and obeys instructions and if they have questions they ask. They don’t keep quiet and if they have challenges, they get back to the doctor almost immediately either via phone, via text or they send somebody. (HCW, East Africa)*

Conversely, PLHIVs forgetting to carry their clinic cards, negotiating for a preferred date for a scheduled CD4 test, reporting an inability to use condoms as directed, using alcohol, missing clinic appointments and improper pill adherence were perceived to be challenging the clinician’s authority and not committed to treatment. In some countries, a disciplinary committee met non-conforming PLHIVs, issued warnings and recommended corrective actions, which PLHIVs were required to follow. Reviews of PLHIV conduct took place after a period of time and directives were then adjusted accordingly. The disciplinary actions in some cases included patients being given more frequent appointment times, which led some PLHIVs to disengage with care.

In contrast, some PLHIVs interpreted the ‘abuse’ and the disciplinary actions taken by the providers as sign of heightened care and concern for their health, which encouraged conformity to the engagement rules.

*If we fail to come on an appointment date and he doesn’t say something, just know that the person is not good. But if he shouts at you it’s when we know that the person is good. (PLHIV, on ART, Southern Africa)*

### The influence of significant others on patients’ attitudes towards HIV care and treatment services

Some PLHIVs appeared to be influenced by the attitudes of others, either their friends, family or general community members, with regard to HIV care seeking and attitudes towards providers. In some instances, this led PLHIVs to avoid certain facilities or disengage with care and seek alternatives.

*What people think…is that the health centre we have here is very small…they think that they don’t get help when they go to the health centre [and subsequently do not attend]. (PLHIV, recently initiated on ART, Southern Africa)*

The relatives of deceased PLHIVs also reported diverse experiences with providers. Some described provider interactions favourably, while others reported bitter memories of neglect and indifference that they and their loved ones experienced, which could subsequently affect their own interactions with health services.

*When I called the doctor, the doctor was just quiet, and after he had died, that was the time the doctor came and rushed him to the ICU, and they put him there, there was nothing, that was when I differed with them, ‘you are putting someone who has already died at the ICU, the time I needed your help you refused,’ and they told me ‘Woman, what do you know, we will chase you out.’ (Family member of a deceased PLHIV, East Africa)*

Some PLHIVs sought care at particular facilities based on recommendations of their friends or relatives who had a positive view of the providers within the referral facility.

*My daughter was sickly. She was coughing and when I asked, I was told that she had been taken to a nearby clinic. Then villagers advised me to take her to a neighboring health center, and I took her there and I was given the same coughing drugs. (PLHIV, lost to follow-up, East Africa)*

### The resources available within health services

PLHIVs reported more favourably on facilities where personnel were ‘available’ and ‘accessible’. Timely availability and accessibility conferred a sense of care for their condition and well-being.

*I can say that what they [HCW] do well is that they are always prepared, having the things ready for their people. They really know what people [patients] want and they make sure that they get them. I salute them for that. (PLHIV, on ART, Southern Africa)*

Conversely, insecure medical, clinical and laboratory supplies diminished their trust in the system, including the HCW within such facilities.

*This is because those who offer services are there, but there is nothing to do with those services, because you will find that there are doctors, and there are no gloves, there is no Panadol, so what can they do? Sometimes lack of test kits, sometimes they have come and the test kits are not there for them. (HCW, East Africa)*

Additionally, providers cited ‘tiredness’ and, in some instances, ‘burnouts’ from ‘overwhelming workload’ as barriers to providing quality care and treatment for PLHIV. While some PLHIVs registered concerns that their complaints were not adequately listened to, others appeared sympathetic to the providers’ plight.

*There are times when I could feel for them when we came and saw them that they were too busy and they would tell us to come the following day if we still have our supply of pills. (PLHIV, on ART, Southern Africa)*

## Discussion

Patient–provider interactions are complex, multidimensional and deeply embedded in wider contextual factors and social dynamics. These interactions are best understood in relation to broader notions of power hierarchies present in patient–provider encounters, community experiences and the structural factors limiting many rural health facilities in sub-Saharan Africa. For these reasons, interventions looking to improve patient experiences and treatment adherence outcomes through enhanced patient–provider interactions need to go beyond the existing focus on patient–provider communication strategies towards patient empowerment.^25 26^

Our data suggest that perceived identities and roles of providers within the HIV care system in sub-Saharan Africa can create an environment conducive to asymmetrical relationships between health providers (the knowledgeable healers) and patients (the ignorant sick). In most settings involved in our study, this context positioned providers as ‘rule setters’ to which PLHIVs are obligated to abide by — diminishing opportunities for constructive and connected relationships. As a consequence and resonating with findings from another study in Zimbabwe,^13^ most PLHIVs found themselves at a disadvantage, having to either submit to or defy the domineering attitudes of providers. For patient empowerment to take root, these hierarchical relationships that persist in the provision of medical care by health workers in many of the settings in our study^27 28^ will need to change.

Our data also reveal a ‘submissive’ self-identity and role definition of PLHIVs, which appear to be influenced by their individual interactions with providers and reinforced by the opinions of others in their households and communities. These submissive PLHIVs were often seen as cooperative and generally rewarded with labels of ‘good patients’, whereas more ‘defiant’ PLHIVs were seen as uncooperative and ‘punished’ with labels of ‘bad patients’. Cooperation of PLHIVs was a notion equivalent to patients’ ability to accept conditional ‘rules of engagement’ stated by providers. This led to involuntary conformity among those who were uncooperative, and thus experiences of powerlessness.^25^ Our study suggests that this disconnect ultimately affects the choices, confidence and opportunities for PLHIVs in care. These findings underscore the notion that patient–provider relationships are critical to vulnerable patients who are reliant on the providers’ competence, skills and goodwill.

The disciplinary actions applied to ‘non-conforming patients’ were enactments of authority to ensure compliance. While effective for some, they fail to recognise the diversity and autonomy of PLHIVs.^29^ Some PLHIVs were able to recognise factors beyond the control of providers that hindered access to care, such as workload and lack of resources, to an extent of showing sympathetic attitudes towards their providers. However, overall these disciplinary actions heightened fear, invited defiance and encouraged late presentation to facilities or disengagement in care and treatment.

PLHIV decision-making to seek care, including defining the source of care, was affected by the attitudes and beliefs of significant others through either active or passive persuasion. Additionally, perceptions of facility resource(s) and capacity affected PLHIVs’ attitudes towards care and providers in general, although value may vary between patients and providers.^30^ Providers in facilities with limited resources and capacity were seen as less helpful towards PLHIVs, and as such were less desired and sought out. Some providers felt overwhelmed with high volumes of patients eliciting empathy from the PLHIVs.^31^ These findings resonate with literature from other studies suggesting that structural elements affect therapeutic relationships, and that organisational and system factors promote continuity in clinical relationships and can strengthen care.^32^

The strength of this study is our ability to document the accounts of PLHIVs with different diagnosis and treatment histories, including those lost to follow-up, family members of people who died due to HIV, and HCWs, and to compare these across several settings. However, when interpreting these findings, it is important to consider possible social desirability bias in the participant’s views.

## Conclusion

Our findings point to the need for greater efforts to enhance patient–provider dialogue and ongoing supportive relationships that promote patients’ confidence and engagement in long-term HIV care and improve ART adherence. Asymmetrical power dynamics in the patient–provider relationship between PLHIVs and providers can affect engagement with care. Interventions should support health providers to empower patients to negotiate care and make decisions about their own health. Such efforts may help improve dynamics in patient–provider relationships, and ultimately increase retention in care. We recommend interventions targeted at restoring the disrupted identities of PLHIVs and reducing the social distance between providers and care seekers in order to promote patient engagement and improve ART adherence.

**Key messages**

- Conformity or resistance to the adoption of a compliant patient persona was affected by an array of inter-related contextual factors and dynamics.
- The perceived roles and identities of the providers and patients influence their interactions and impact on subsequent engagement in care.
- Interventions to improve patients’ outcomes through enhanced patient–provider interactions should consider the role of social dynamics operating at individual, interpersonal and structural levels.
